# Cross-Sectional and Prospective Associations between Physical Activity and C-Reactive Protein in Males

**DOI:** 10.1371/journal.pone.0125984

**Published:** 2015-05-11

**Authors:** Aírton J. Rombaldi, Lúcia C. Pellanda, Renata M. Bielemann, Denise P. Gigante, Pedro C. Hallal, Bernardo L. Horta

**Affiliations:** 1 Post-Graduate Program in Physical Education. Federal University of Pelotas, Pelotas, Brazil; 2 Federal University of Health Sciences of Porto Alegre, Porto Alegre, Brazil; 3 Institute of Cardiology of Rio Grande do Sul, Porto Alegre, Brazil; 4 Post-Graduate Program in Epidemiology. Federal University of Pelotas, Pelotas, Brazil; University of New South Wales, AUSTRALIA

## Abstract

**Background:**

There is conflicting evidence about the association between physical activity and inflammatory markers. Few prospective studies are available, particularly from low and middle-income countries. This study was aimed at assessing the cross-sectional and prospective associations between physical activity and C-reactive protein (CRP) levels in males belonging to the 1982 Pelotas (Brazil) Birth Cohort Study.

**Methods:**

The sample comprised 2,213 males followed up at the ages of 18 and 23 years. We performed high sensitivity CRP assays; we used a cut-off of 3 mg/L in categorical analyses. We measured physical activity by self-report at ages 18 and 23 years. Body mass index and waist circumference were studies as possible mediators.

**Results:**

CRP levels above the 3mg/L cut-off were found in 13.3% (95%CI: 11.7; 14.8) of the individuals. We found no evidence for an association between physical activity (leisure-time or all-domains) and either continuous (geometrical mean) or categorical CRP. We confirmed these null findings in (a) prospective and cross-sectional analyses; (b) trajectories analyses.

**Conclusions:**

There was no association between CRP levels and physical activity levels in early adulthood in a large birth cohort. Little variability in CRP at this early age is the likely explanation for these null findings.

## Background

Chronic low-grade inflammation is associated with many chronic diseases, including atherosclerosis, obesity, metabolic syndrome and diabetes[1, 2]. Blood levels of the inflammatory acute-phase reactant C-reactive protein [CRP]) are a marker of inflammation widely used in clinical practice[3].

Physical activity may be an important protective factor for low-grade inflammation, among several other lifestyle-related behaviors [4–6]. CRP levels have been shown to be inversely related to moderate to vigorous intensity physical activity practice in many settings[7]. In experimental and cross-sectional studies, physical activity leads to reduction in inflammatory markers with physically active individuals showing lower levels of inflammatory markers as well as lower global cardiovascular risk[8]. However, there is still controversy regarding the association between physical activity and inflammation, with some clinical studies showing no anti-inflammatory effects[9, 10] and few studies evaluating prospectively the influence of physical activity on CRP levels[11].

It remains to be established prospectively how the inflammatory response to physical activity occurs in longer periods of follow-up, especially in healthy young adults, in whom chronic disease is still not present as a confounding factor. It is also unknown how stability or changes in physical activity habits during early adulthood impact this association. Since compliance to long intervention protocols is difficult to attain, this questions may best be answered by long-lasting cohort studies. Finally, population-based cohort studies may evaluate general physical activity patterns as adopted by individuals in “real life”, as opposed to highly structured exercise programs in randomized clinical trials, often performed in selected samples and ideal conditions that are difficult to reproduce in large scale.

Thus, the aim of this study was to assess the cross-sectional and prospective associations between physical activity levels at 18 and 23 years of age, and CRP levels at 23 years in healthy males belonging to the 1982 Pelotas (Brazil) Birth Cohort, in order to describe the low-grade inflammatory response to physical activity in these different moments. Additionally, we sought to evaluate the differences between maintenance or changing in physical activity patterns and CRP levels.

## Methods

The 1982 Pelotas (Brazil) Birth Cohort Study is being conducted in Pelotas, a southern Brazilian city of ~340,000 inhabitants. In 1982, all maternity hospitals in the city were visited daily and the newborns identified. The cohort enrolled 5914 live births to mothers who lived in the urban area of the city that year (population in 1982: 214,000 people), representing over 99% of total births. The cohort has been followed up numerous times throughout the years. The study methodology has been described elsewhere[12].

For the present analyses, we used information from males interviewed specifically in two follow-ups. Firstly, from July to September 2000, all male subjects were identified during the Army medical examination when the cohort members were at a mean age of 18.2 years. The subjects were asked about physical activity and other health-related topics, such as smoking, diet and alcohol intake. In this follow-up, 2,250 of the 3,037 males of the cohort were interviewed. Five years later, from October 2004 to August 2005, a census was carried out in the city, in search of subjects belonging to the cohort, and 2,213 male subjects were interviewed. After taking into account known deaths (2000: 143; 2004–5: 159), this represented follow-up rates of 79% and 78%, respectively. The study was approved by the Ethics Committee of the Medical School of the Federal University of Pelotas. Written informed consent was obtained from every subject prior to the interviews and blood sample collection.

Of the interviewed males, 1,918 (87%) provided a blood sample. Non-fasting blood was collected by venous puncture. High sensitivity CRP assays were performed using the automated DPC (Siemens) Immulite chemiluminescent immunoassay (Los Angeles, CA, USA). The intra- and inter-assay coefficients of variation were 10 and 7%, respectively. Samples with results below the assay sensitivity threshold, which registered as <0.1 mg/L, were converted to 0.05 mg/L for statistical analysis. CRP levels above 3 mg/L were considered high[13].

Physical activity was measured using different questionnaires at 18 and 23 years of age. However, both questionnaires allowed measurements of minutes spent in physical activity per week. In 2000, subjects were asked about the practice, frequency and duration of sports and exercises in a usual week anywhere (e.g. gym, sports club, household, school, commuting to work, etc.). In the 2004–5 follow-up, we used the long version of the International Physical Activity Questionnaire (IPAQ). The long version of the IPAQ evaluates walking, moderate-intensity and vigorous-intensity physical activity according to frequency and duration in four domains—occupational, household, leisure time and commuting[14]. The time spent in vigorous-intensity physical activity was multiplied by two.

From the data on physical activity at 18 and 23 years-old, we estimated changes in the five-year period, and subjects were classified in one of the following categories: inactive at both ages, active only at 18 years-old, active only at 23 years-old and active at both ages. We considered as “active”, males who achieved the current physical activity recommendation for adults of at least 150 minutes/week of moderate-to-vigorous physical activity[15]. To evaluate possible differences in the effect according to the domains of physical activity, we analyzed physical activity at 23 years using information from the four domains and leisure-time only.

Subjects were also inquired about current smoking, economic status (based on household assets, having a full-time maid, and head-of-family`s schooling, according to the Brazilian Association of Research Institute criterion- ABEP) and skin color (evaluated by self-report in 2004–5). Other variables previously collected were considered as potential confounders: monthly family income at birth and household assets index at 2 years (obtained through factor analysis and based on the ownership of household goods).

Mediating variables evaluated in this study were waist circumference and body mass index (BMI). Waist circumference was measured using a flexible tape (Mabbis) with an accuracy of 1 mm at the narrowest part of the trunk. Standing height was measured to the nearest 1 mm with subjects barefoot using a stadiometer (CMS); the subjects were weighed to the nearest 100 g in their underpants using an electronic scale (SECA–UNICEF). Weight was divided by the square of height in meters to calculate BMI.

Analyses were performed with Stata 12 software (StataCorp, College Station, TX, USA). CRP levels were natural log-transformed due to the skewed distribution. Unadjusted and adjusted analyses were performed using linear regression. Due to log transformation, the results for beta coefficients are shown in exponential function and representing a multiplicative relationship. Results are interpreted as the % change in CRP levels associated with one unit change in the independent variable. The relative risk of each physical activity measurement on CRP>3 mg/L (high CRP level) was determined with 95% confidence interval by Poisson regression with robust adjustment of the variance[16]. In addition, minutes/week spent in physical activity at 18 and 23 years were described according to CRP levels in tertiles with statistical significance tested by analysis of variance. The potential confounders were: skin color, monthly family income at birth, household assets index at 2 years, current economic status and current smoking (model 2). BMI (model 3) and waist circumference (model 4) were tested one at a time as possible mediating factors. The significance level was set at 5%.

## Results

A description of males belonging to the 1982 Pelotas Birth Cohort is presented in the Table 1. Most subjects reached 150 minutes per week at 18 or 23 years (55.4% and 92.2%, respectively). One in two males was active during leisure-time at 23 years. A high CRP level was found in 13.3% (95%CI: 11.7; 14.8) of the participants.

**Table 1:** Description of males from the 1982 Pelotas Birth Cohort followed-up at 23 years of age.

Variable	*N*	Mean (SD)	Prevalence (CI_95%_)
**Smoking at 23 years**	2213		27.6 (25.7; 29.5)
**BMI≥25kg/m** ^**2**^	2206		30.6 (28.7; 32.5)
**Waist circumference** (cm)	2205	80.9 (10.1)	
Physically active at 18 years (Total PA)*	2237		55.4 (53.4; 57.5)
Physically active at 23 years (Total PA) *	2212		92.2 (91.1; 93.3)
Physically active at 23years (LTPA)**	2213		50.7 (48.6; 52.8)
**C-reactive protein** (mg/L)^***^	1839	0.74 (3.77)	
**C-reactive protein > 3 mg/dL**	1839		13.3 (11.7; 14.8)

*Total PA—all 4 domains of physical activity; at least 150 minutes/week of moderate-to-vigorous physical activity.

** LTPA—Leisure-time physical activity; at least 150 minutes/week of moderate-to-vigorous physical activity.

^***^ Geometric mean.

Table 2 shows the association between physical activity measurements at 18 and 23 years in quartiles and log-transformed CRP level at 23 years using four different statistical models. Total physical activity at 18 and 23 years was not associated with CRP level at 23 years. Coefficients of association using total physical activity at 18 and leisure-time physical activity at 23 years were, in general, close to the null value, whereas males in the second and third quartile showed on average 19% lower CRP levels than subjects in the first quartile. However, the 95% confidence intervals included the null value (95% confidence intervals: 0.65; 1.01 and 0.66; 1.01—respectively).

**Table 2:** Physical activity at 18 and 23 years and CRP level in young males from Brazil.

Physical activity measurement	Log-transformed C-reactive protein							
	Crude (n = 1739)		Model 2^a^ (n = 1113)		Model 3^b^ (n = 1111)		Model 4^c^ (n = 1111)	
	β coefficient (95%CI)	p	β coefficient (95%CI)	p	β coefficient (95%CI)	p	β coefficient (95%CI)	P
**Total physical activity at 18y** (min/week-quartiles)		0.176		0.982		0.942		0.977
1^st^	Ref.		Ref.		Ref.		Ref.	
2^nd^	1.11 (0.94; 1.32)		1.01 (0.82; 1.25)		1.02 (0.83; 1.24)		1.04 (0.85; 1.27)	
3^rd^	1.19 (0.99; 1.42)		1.03 (0.83; 1.28)		0.96 (0.78; 1.18)		1.00 (0.81; 1.23)	
4^th^	1.19 (0.99; 1.44)		1.05 (0.83; 1.31)		0.99 (0.79; 1.22)		1.03 (0.83; 1.28)	
**Total physical activity at 23y** (min/week-quartiles)		0.535		0.208		0.373		0.557
1^st^	Ref.		Ref.		Ref.		Ref.	
2^nd^	0.87 (0.72; 1.05)		0.81 (0.65; 1.01)		0.84 (0.68; 1.04)		0.87 (0.71; 1.08)	
3^rd^	0.93 (0.78; 1.12)		0.81 (0.66; 1.01)		0.86 (0.70; 1.05)		0.88 (0.71; 1.08)	
4^th^	0.94 (0.80; 1.12)		0.87 (0.70; 1.07)		0.89 (0.73; 1.09)		0.91 (0.74; 1.11)	
**Leisure-time physical activity at 23y** (min/week-quartiles)		0.823		0.662		0.402		0.574
1^st^	Ref.		Ref.		Ref.		Ref.	
2^nd^	0.95 (0.75; 1.21)		1.14 (0.85; 1.53)		1.19 (0.90; 1.58)		1.19 (0.89; 1.57)	
3^rd^	1.03 (0.88; 1.21)		0.96 (0.78; 1.17)		0.95 (0.78; 1.15)		0.97 (0.80; 1.18)	
4^th^	1.05 (0.90; 1.22)		0.97 (0.80; 1.16)		0.96 (0.80; 1.15)		0.99 (0.83; 1.19)	

CRP—C-reactive protein

^a^ Adjusted for skin color, family income at birth, household assets index at 2 years, current economic status and current smoking

^b^ Adjusted for ^a^ + BMI

^c^ Adjusted for ^a^ + waist circumference.

**Fig 1:**
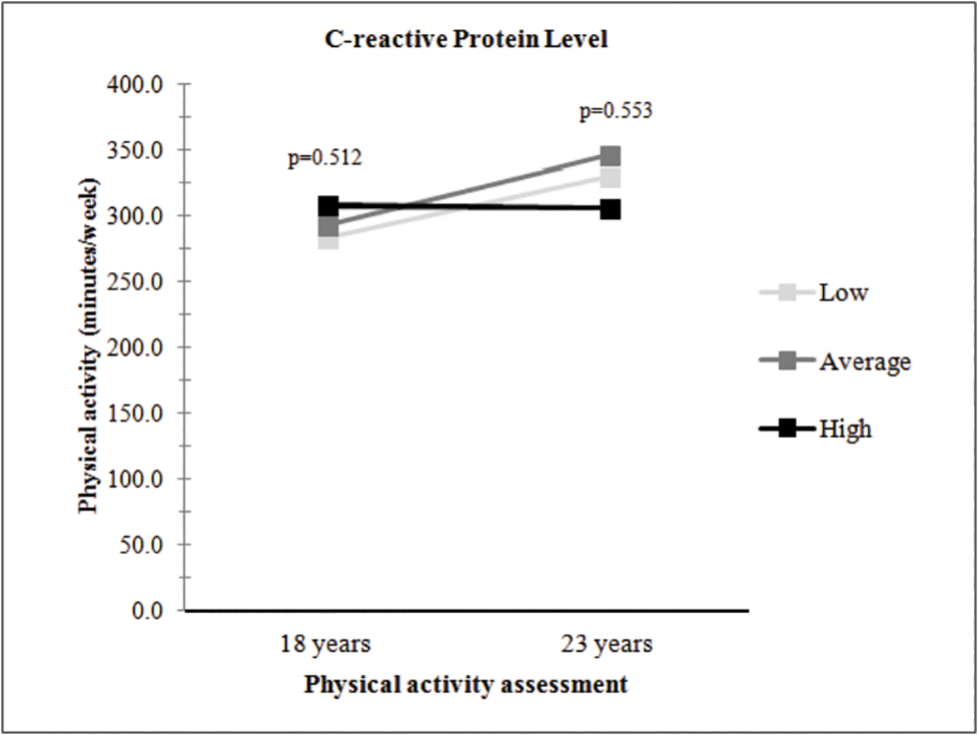
Physical activity at 18 and 23 years according to tertiles of C-reactive protein level in young male adults from the 1982 Pelotas Birth Cohort at 23 years.

A description of physical activity levels according to tertiles of CRP at 18 and 23 years is shown in Fig 1. Time spent in physical activity at 18 years was statistically similar in all tertiles. However, males in the highest tertile of CRP at 23 years spent around 50 minutes less time in physical activity than males in the lowest tertile of CRP.

Table 3 shows variation in (a) all-domains physical activity at 18 and 23 years; (b) all-domains physical activity at 18 years and leisure-time physical activity at 23 years and log-transformed CRP levels at 23 years. In unadjusted analysis, active males at 18 years showed higher CRP levels five years later, when compared to inactive males at both periods. However, there was no statistical significance in adjusted analyses. Likewise, active males at 18 years and during leisure-time at 23 years also had higher CRP levels at 23 years in the unadjusted analysis, but this difference was not statistically significant in adjusted analysis. After adjustment for possible mediators (models 3 and 4), no differences were found. The same null results were found using these exposures in association with the risk of a high CRP level >3 mg/L at 23 years (Table 4).

**Table 3:** Changes on physical activity from 18 to 23 years and CRP in males from Brazil.

Variables	Log-transformed C-reactive Protein (mg/L)^*^							
	Crude (n = 1739)		Model 2^a^ (n = 1113)		Model 3^b^ (n = 1111)		Model 4^c^ (n = 1111)	
	β (95%CI)	p	β (95%CI)	p	β (95%CI)	p	β (95%CI)	P
**Total PA at 18 and 23 years**		0.071		0.778		0.878		0.981
Inactive 18 and 23	Ref.		Ref.		Ref.		Ref.	
Active 18; Inactive 23	1.65 (1.03; 2.62)		1.14 (0.66; 1.96)		0.99 (0.58; 1.67)		1.03 (0.61; 1.75)	
Inactive 18; Active 23	1.10 (0.80; 1.52)		0.92 (0.63; 1.36)		0.89 (0.61; 1.30)		0.96 (0.65; 1.39)	
Active 18 and 23	1.22 (0.89; 1.68)		0.96 (0.66; 1.41)		0.88 (0.61; 1.28)		0.97 (0.67; 1.41)	
**PA at 18 and LTPA at 23 years**		0.176		0.765		0.894		0.957
Inactive 18 and 23	Ref.		Ref.		Ref.		Ref.	
Active 18; Inactive 23	1.17 (0.98; 1.41)		1.08 (0.86; 1.37)		1.00 (0.80; 1.25)		1.03 (0.83; 1.29)	
Inactive 18; Active 23	1.09 (0.90; 1.33)		0.96 (0.76; 1.22)		0.95 (0.75; 1.19)		0.97 (0.77; 1.23)	
Active 18 and 23	1.19 (1.01; 1.41)		0.99 (0.80; 1.22)		0.94 (0.77; 1.15)		0.98 (0.80; 1.20)	

CRP—C-reactive protein

Total PA—all 4 domains of physical activity; LTPA—Leisure-time physical activity at 23 years

^*^Logarithm transformation was used

^a^ Adjusted for skin color, family income at birth, household assets index at 2 years, current economic status and current smoking

^b^ Adjusted for ^a^ + BMI

^c^ Adjusted for ^a^ + waist circumference.

**Table 4:** Changes on physical activity from 18 to 23 years high CRP level in Brazilian males.

Variables	C-reative protein > 3 mg/L			
	Crude	Model 2^a^	Model 3^b^	Model 4^c^
	RR (95%CI)	RR (95%CI)	RR (95%CI)	RR (95%CI)
**Total PA at 18 and 23 years**				
Inactive 18 and 23	Ref.	Ref.	Ref.	Ref.
Active 18; Inactive 23	1.87 (0.76; 4.62)	1.24 (0.39; 3.93)	1.05 (0.31; 3.50)	1.11 (0.34; 3.63)
Inactive 18; Active 23	1.21 (0.58; 2.53)	1.29 (0.54; 3.05)	1.36 (0.52; 3.58)	1.44 (0.54; 3.78)
Active 18 and 23	1.41 (0.69; 2.91)	1.25 (0.53; 2.92)	1.27 (0.48; 3.31)	1.37 (0.52; 3.60)
**PA at 18 and LTPA at 23 years**				
Inactive 18 and 23	Ref.	Ref.	Ref.	Ref.
Active 18; Inactive 23	1.27 (0.90; 1.79)	1.11 (0.70; 1.75)	1.02 (0.66; 1.60)	1.06 (0.68; 1.65)
Inactive 18; Active 23	0.92 (0.62; 1.39)	0.90 (0.54; 1.50)	0.94 (0.57; 1.54)	0.96 (0.58; 1.57)
Active 18 and 23	1.09 (0.78; 1.52)	0.85 (0.54; 1.31)	0.85 (0.55; 1.30)	0.88 (0.57; 1.37)

CRP—C-reactive protein.

Total PA—all 4 domains of physical activity; LTPA—Leisure-time physical activity at 23 years

^a^ Adjusted for skin color, family income at birth, household assets index at 2 years, current economic status and current smoking

^b^ Adjusted for ^a^ + BMI

^c^ Adjusted for ^a^ + waist circumference.

## Discussion

In this analysis of the 1982 Pelotas (Brazil) birth cohort, CRP levels were not associated with physical activity levels in early adulthood. Although reduction in inflammation has been investigated as a possible mechanism for the benefits of physical activity in reducing cardiovascular and other chronic disease outcomes, studies have been controversial regarding the anti-inflammatory effects of physical activity.

There are many possible explanations for our findings, including little variability in CRP at this young age, the small interval between the two measurement periods (five years only) and the use of self-reported instead of objectively measured physical activity. It is also possible that the association described in older adults is still not present in youth. At this age, chronic disease is still not present as a confounding factor, and the intensity of inflammation may be lower and homogeneous between subjects, making it more difficult to identify small differences. In our study, only around 13% of the participants showed high CRP levels whereas in the EPIC-Norfolk study, for example, a third of the adults with age ranging from 40 to 79 years had serum CRP levels above 3 mg/L[17]. Accordingly, many studies in children and young adults tend to show no association, in agreement with our findings[18], while studies in older adults show an inverse association between regular physical activity and levels of inflammatory markers[8, 19–22]. The Whitehall II cohort study, for example, evaluating adults with a mean age of 49.2 years, showed that those who were physically active at baseline showed lower CRP and interleukin-6 (IL-6) levels both at baseline and after 10 years of follow-up [20]. On the other hand, a study with 205 children and young adults showed that fitness level was inversely correlated with CRP levels, especially in boys. In this study, fitness was evaluated by treadmill testing, and there was a great age range, from 6 to 24 years old[23].

As well as the overall lower levels of inflammation, individuals in this age group tend also to be more homogeneously active, since physical activity decreases with increasing age[24]. In our study, most subjects reached the current guidelines of at least 150 minutes/week of physical activity, particularly at 23 years of age, when all four domains of adult activity were taken into account, while in the Whitehall II cohort study only half of participants were considered active in overall physical activities [20].

Differences in patterns of physical activity must also be taken into account. Intensity, duration and type of activity, and individual characteristics such as race, gender and disease status, may all play a role in modulating these responses[25–27]. Socioeconomic factors may also influence the type and intensity of exercise. Most cohort studies are from highly income countries, where most physical activity occurs during leisure, being vigorous and time-limited. In low and middle-income countries, composition of physical activity may differ, being mostly related to work and transportation, usually with longer duration and lower intensity[28]. These differences may make comparisons across studies more complex, but also may offer interesting clues about the type and intensity of exercise that would be more beneficial[29–31].

The type of study is also important when considering controversial results. Most beneficial effects associated with physical activity are described to be result of chronic training adaptations. Thus, short term randomized clinical trials with highly structured exercise programs may not capture the long-term effects of more ample physical daily activity in “real life”, which may explain the differences between randomized and observational data.

When we consider the variability of CRP, the reliable biologic variability data that represent a fundamental prerequisite for the correct definition of specific analytical goals for imprecision, bias and total allowable error, permitting the safe application of laboratory measurements in clinical setting is lacking. A recent report concluded that there is a paucity of robust data on biologic CRP variability[32]. Moreover, the CRP cutoff point used to identify individuals at high risk was based on data from European and European-American populations, where there is high prevalence of adults with ≥ 3mg/L. The use of these cutoffs for other groups in not well-established[33]. Other possible explanation for the lack of association observed in the present study is that physical activity may reduce CRP in patients with elevated levels of these markers, but exert little influence in individuals with normal levels. In a study with diabetic patients in a 18 years- follow-up, physical activity reduced mortality significantly in patients with elevated hs-CRP levels (>.3 mg/L), but not in patients with levels below 3 mg/L[34].

There are periods in growth and development described as critical. Beneficial or deleterious stimuli occurring during these specific periods may lead to permanent health effects. For example, exercise during pregnancy and early childhood appears to have long-term effects over growth mediators and stress/ inflammatory factors[35]. We may hypothesize that these effects of programming, being mediated mostly by chronic, bland inflammation, take a long time to manifest[36]. Thus, our findings reflect a possible absence of association in early adulthood, but it is possible that this association will manifest later in life[37]. This is important to add to the understanding of the complex associations between exposures and outcomes across the life course. Chronic inflammation is involved in the pathogenic pathways of atherosclerosis and insulin resistance, and the protective effect of physical activity has been linked to its anti-inflammatory effects. These effects may be mediated by a reduction in visceral fat mass, but may also occur independently of adiposity, via the increase in circulating anti-inflammatory cytokines including interleukin (IL)-1 receptor antagonist and IL-10[38]. These manifestations differ according to the type and duration of physical activity, and may be more apparent after prolonged periods of regular activity. With the continuation of the cohort, it may be possible to observe lower CRP levels in individuals that who remained active in both 18 and 23 years, using this information as a proxy of regular activity in a 5-year period, thus the importance of describing the present data.

One important limitation of our study that merits discussion is the use of questionnaires to measure physical activity. These instruments are useful to compare populations in epidemiological studies, and to screen patients in clinical settings. However, they may be insufficient for more detailed studies of associations with biological outcomes. More complex methods to measure physical activity may be more accurate, but are often unfeasible in large studies[39]. In addition, with a power of 80% we were able to identify only statistically significant reduction on CRP levels above 17% (for physical activity at 18 and 23 years during leisure-time) and 35% (for physical activity in four domains at 23 years) between active and inactive groups. However, the differences on CRP levels between groups of physical activity were of lower magnitude, and therefore, non-significant.

In conclusion, there was no association between CRP levels and physical activity levels in two different moments of early adulthood in a large birth cohort. These findings may be important in the future to identify the moment when this associations begin to manifest, helping to establish optimal moments for intervention.
